# Microstructural and Mechanical Implications of Microscaled Assembly in Droplet-based Multi-Material Additive Manufacturing

**DOI:** 10.3390/polym9080372

**Published:** 2017-08-18

**Authors:** Sofiane Guessasma, Hedi Nouri, Frederic Roger

**Affiliations:** 1 INRA, UR1268 Biopolymères Interactions Assemblages, F-44300 Nantes, France; 2 IMT Lille Douai, Institut Mines-Télécom, Polymers and Composites Technology & Mechanical Engineering Department, 941 rue Charles Bourseul, 59508 Douai, France; hedi.nouri@imt-lille-douai.fr (H.N.); frederic.roger@imt-lille-douai.fr (F.R.)

**Keywords:** droplet-based additive manufacturing, X-ray micro-tomography, multi-material print, interface properties

## Abstract

To reveal the potential and limits of multi-material three-dimensional (3D) printed parts in droplet-based additive manufacturing, a study combining tensile experiments and 3D imaging technique is proposed. A polymeric composite structure made of acrylonitrile butadiene styrene and thermoplastic polyurethane is manufactured using a two extrusion head printer. The quality of the interface between the two thermoplastics is quantified by adjusting the number of intertwining droplets at the interface. Tensile experiments assisted with digital image correlation are performed with two-interface orientation to discriminate shearing and traction at the interface. The 3D imaging results, which are based on X-ray micro-tomography, show the distinct features of droplet-based additive manufacturing in terms of porosity content and connectivity. Interface properties are found to control, in an incomparable way, the mechanical response. It is found that the interface quality is determinant for enhancing the ultimate performance whereas the interface orientation is found to be the perfect leverage for varying the slope of the linear part.

## 1. Introduction

Additive manufacturing (AM) is a new route for the production of complex parts from three-dimensional digitalised models [1,2]. The fabrication cycle for a typical design is a contrast between flexibility and printing time [3,4]. On one hand, the conversion of any CAD model into series of tool paths boils down to a simple matter of hashing or slicing_ENREF_2 the design surfaces with limited tool dependency [5,6,7]. On the other hand, deciding at a fine scale where to print the material turns out to be a slow process that leads to productivity problems [3]. Despite limits related to large-scale production, a growing interest is being witnessed for AM technologies [5]. Such interest has been gained from key fields [6] such as the automotive [7], aerospace [8], bioengineering [9], and civil engineering [10] industries. Some of the arguments in favour of further development of AM processes include the possibility of product customisation [4], the CAD-to-part concept [6], the short time-to-market [11], the remote production perspective [12], resource optimisation with a positive environmental footprint [13], the versatility of techniques and materials [1,5,14] for a wide spectrum of applications [7,8,9], and the rapid maturity of AM techniques [6]. The arguments against are problems of accuracy [15,16,17], low production rates [6], difficulty of performance tailoring (anisotropy, defects, durability) [15,16], unsatisfying finishing state (including roughness and residual support) [17], and lack of regulation and standards [5,18].

In addition, one major limitation of AM processes that justifies extensive work on the subject is the limited size of parts that can be printed. This limit can be explained by several factors such as the difficulty in maintaining steady thermal behaviour during the processing of large parts. This factor is critical because there is a strong correlation between the structural integrity of the part and its thermal history. This is demonstrated, for instance, by the genesis of the inter-filament bond strength [19]. Another factor is the kinematics of printing, which needs to balance accuracy and rate. For AM processes based on fused deposition, this means difficulty in maintaining accuracy in positioning the printing nozzle when displacement rates are increased. For powder-based processes, a dwelling time at a particular spot is needed to achieve local powder fusion and avoid a balling effect. In metal additive manufacturing, this is perfectly illustrated by the negative correlation between the material density and the scanning rate [20].

Most of the commercial solutions available under an affordable budget exhibit a limited printing volume of less than 20 × 20 × 20 cm^3^. The design of larger parts comes at a higher cost and only a few professional solutions are available. Major acting companies are now directing their research and development (R&D) activity towards concepts of infinite-build printers, which are believed to be the future solutions for demanding markets such as aerospace and civil engineering [6]. These solutions are, however, still out of reach.

Another way of achieving large prints is to assemble smaller printed parts using available three-dimensional (3D) printers without investing in expensive solutions. To be effective, assembly of printed parts needs to be performed with care to avoid risk of failure at junctions. This work attempts to understand the limits and opportunities of assembling 3D parts obtained by multi-head printers. We focus on the droplet-based additive manufacturing of a 3D printed plastic composite. This manufacturing method is selected because it allows a control of the quality of the interface by adjusting the intensity of material overlapping at the interface. The sensing of the interface quality is permitted in this process thanks to the adjustment of the number of intertwining droplets at the interface. In addition, we introduce another control parameter, which is the orientation of the interface. This parameter allows varying the balance between shearing and normal traction when the printed two-phase material is subject to a tensile loading. Finally, X-ray micro-tomography is used to achieve the microstructural interpretation of the observed response. This is the method of choice to reveal the 3D microstructure of printed parts [21]. Thanks to the principle of density contrast between phases, phase distribution, microscopic defects and assembling efficiency can be explored. In this study, porosity characteristics measured from X-ray micro-tomography are related to both the mechanical response of the polymeric composite and the interfacial behaviour.

## 2. Experimental Layout

The feed materials are two thermoplastics, namely acrylonitrile butadiene styrene (ABS Terluran GP35 Green) and thermoplastic polyurethane (TPU Elastollan EC78A15,) purchased from BASF GmbH, Ludwigshafen, Germany. Despite the incomplete set of mechanical properties provided by the manufacturer (Table 1), it is clear that ABS is stiffer and has greater resistance compared to TPU. The studied composite offers a large Young’s moduli ratio of 4.36 in favour of ABS.

**Figure 1:**
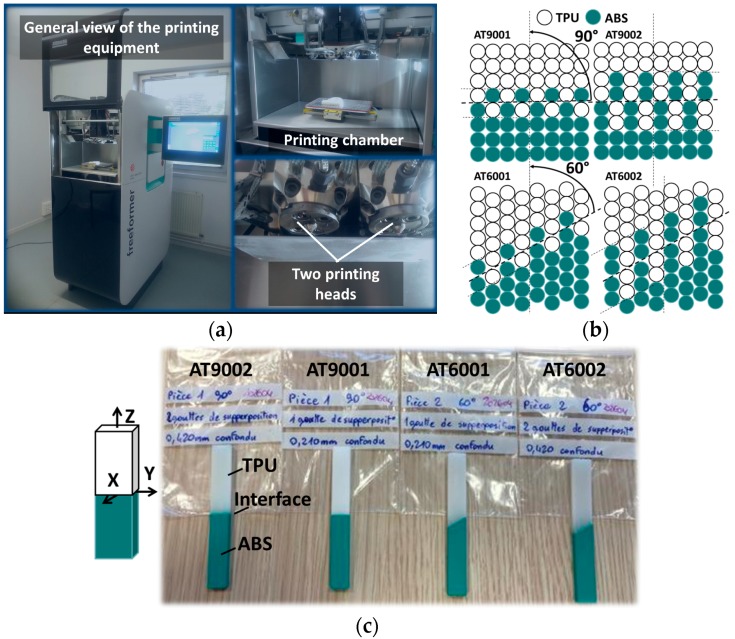
(**a**) Overview of the additive manufacturing equipment based on plastic droplet deposition; (**b**) Illustration of droplet-based structures showing different interfacial configurations. A refers to ABS, T refers to TPU, 60 and 90 refer to the assembly angle with respect to main specimen direction, and 01 and 02 are the number of intertwining droplets at the interface; (**c**) As-printed 3D parallelipipedic slabs (70 × 10 × 4 mm^3^) using multi-material printing solution.

Tensile properties of Elastollan and Terluran in Table 1 are determined according to the norms DIN 53504 and ISO 527–1/–2, respectively. Parallelipipedic specimens of ABS-TPU composite are printed using multi-material 3D printer (Arburg Plastics Freeformer from Arburg, Loßburg, Germany). This equipment uses the principle of high frequency (60 to 200 Hz) plastic droplet deposition to allow printing multi-material features with two extrusion heads (Figure 1a).

A printing nozzle of 150 µm in diameter is used and the layer thickness is adjusted to 0.21 mm with 100% infill. Regular designs consist of half ABS–half TPU specimens intersecting at an angle of 60° and 90° with respect to the tensile direction (Figure 1b,c). Design dimensions are as follows: length = 70 mm, width = 10 mm, thickness = 4 mm. The building direction is aligned with the specimen thickness. With a typical weight of 3.41 g per sample, the overall density is 1.22 g/cm^3^. The first configuration with an interface inclination of 60° is selected to achieve a mixture of tension and shearing at the interface, while the second one is meant to measure only the normal interfacial resistance. In addition, the printing configuration is adapted to allow one or two intertwining droplets at the interface for each interface orientation (Figure 1b). The resulting interphase zone has a typical thickness of 0.21 and 0.42 mm, respectively for one and two intertwining droplets. The total number of configurations combining interface orientations and intertwining droplets is four. The adopted sample nomenclature ATXXYY intuitively includes the used feed materials (A: ABS, T: TPU) and the printing conditions (XX: angle, YY: number of intertwining droplets).

**Figure 2:**
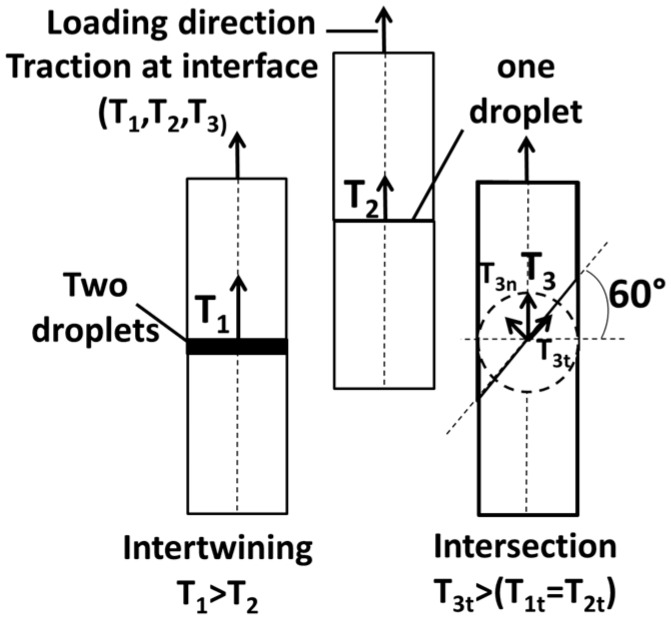
Illustration of the expected effects from varying the number of intertwining droplets and the intersection angle at the interface.

Figure 2 illustrates the expected effect from the former configurations. When increasing the number of droplets, the loading transferred to the interface results in a larger traction, and greater interfacial stiffness, which ideally reaches infinity if a zero displacement jump at the interface is obtained (case of perfect interface [22]). The expected effect is a stronger interface that allows a better transfer of the traction and displacement. When the intersection angle is 90°, this means that the loading direction is aligned with the interfacial traction. In this case, the expected interfacial effect is an exclusive normal tension (*T*_1_ = *T_1n_* and *T*_2_ = *T_2n_*, where the index n refers to the normal component of the traction at the interface). The change of the intersection angle to 60° results in a positive shearing component at the interface [23] (*T*_3_ = *T_3n_* + *T_3t_*, where the index t refers to the tangential component of the traction at the interface). This means that the loading response is expected to depend on the interfacial shear stiffness (Figure 2).

The manufacturing process is conducted by printing an external frame prior filling the inner part. Because of the rapid cooling down of the external frame, it is soldered with the rest of the structure by allowing 75% of overlapped droplets. Using a printing rate of 40 mm/s for the first layer and 80 mm/s for the remaining ones, the printing duration per sample is 35 min.

**Figure 3:**
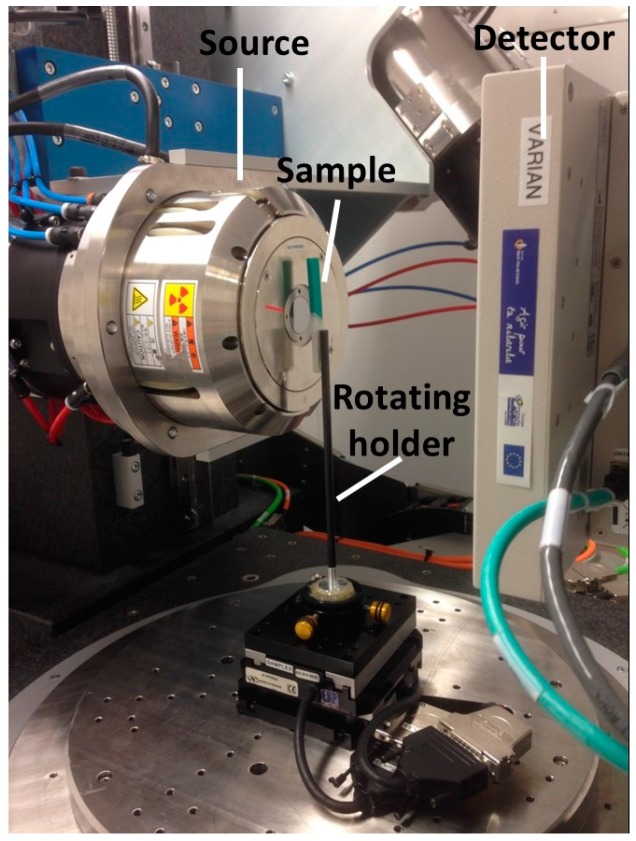
X-ray micro-tomography setup to acquire three-dimensional (3D) microstructural images of multi-material printed slabs.

The microstructural rendering of the droplet-based 3D printing process is evaluated using a 3D imaging technique based on X-ray micro-tomography (Figure 3).

Image acquisition is performed using UltraTom X-ray µ-CT equipment. The acquisition parameters are: 160 kV X-ray source, voltage (60 kV), current intensity (70 µA), varian detector, resolution (1920 × 1536 pixels), voxel size (8.36–10.92 µm), number of radiographic images (1440), sample–source distance (13–17 mm), sample–detector distance (191 mm), continuous mode acquisition, and a reconstruction algorithm using the filtered back-projection method (X-Act software from Rx-Solutions, Raleigh, NC, USA). Because of analysis cost, only one sample per condition is imaged. The acquisition duration varies between 912 and 1362 s.

The data collected are of typical resolutions varying between 0.55 and 0.84 billon voxels, and these correspond to imaged regions of interests having volumes between 4 × 10 × 10 mm^3^ and 5 × 14 × 11 mm^3^.

Image processing is conducted on raw images to highlight different features including porosity and filling characteristics at the TPU/ABS interface. Image operations include dilation, erosion, thresholding, and labelling. In addition, flood filling and wrapping techniques are used to isolate the sample from the background. Statistical analysis related to the sensed defects is performed using more elaborated image analysis protocols. The size distribution of the porosity is determined using a 3D granulometry technique [24]. This protocol is based on a numerical sieving conducted in an iterative way on binary images containing the isolated porosity features. Successive dilation and erosion operations are realised using an octahedral structuring element, for which the size increases by one voxel after the completion of each iteration. The process sieves the porosities depending on their size and continues until no porosities are left in the stack. The former image processing protocol cannot be used to determine the porosity connectivity because sieving disconnects porosity features along the process. To allow a complementary analysis of the porosity connectivity, a 3D labelling technique is used [25]. Starting from the binary image representing the porosity network, voxels belonging to the porosity features are identified by the same label if they share corners or faces (26-connectivity).

**Figure 4:**
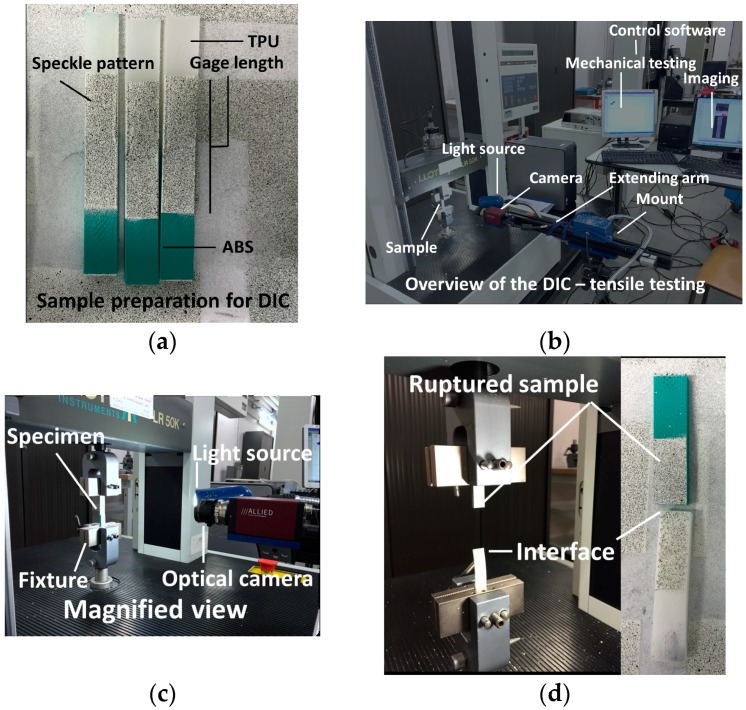
Experimental setup for mechanical testing and Digital Image Correlation (DIC). (**a**) Speckle pattern performed prior to testing; (**b**) overview of the testing equipment; (**c**) magnified view showing the DIC coupled to tensile testing; (**d**) evidence of interfacial rupture.

Uniaxial testing is performed using Lloyd universal testing machine with a load cell capacity of 10 kN (Figure 4). Testing of four samples is sufficient to allow an accurate measurement of tensile performance. As shown in [26], the difference between replicates can be as small as 1% (difference between mechanical response measured from the area under the curve). In the current study, we achieve a standard deviation between 1.5% and 5.4% depending on the printing condition. This represents an accuracy of 0.45 MPa in strength determination.

Tensile loading is based on a displacement control with a constant rate of 5 mm/min. The gage length is adjusted between 41 and 42 mm (Figure 4a). Optical image recording of the sample deformation is performed simultaneously with the tensile loading to allow local deformation to be captured (Figure 4b,c). To do so, digital image correlation equipment from Correlated Solutions company (Irmo, SC, USA) is used. The setup consists of a 4-megapixel (2000 × 2000 pixels) camera, a light source and a control software (VIC-2D software). The optical recording is conducted with a frame rate of 5 fps (frame per second). The recording is centred on a region of interest (ROI) of typical dimensions of 100 × 30 mm². The surface exposed to optical recording is coated with a layer of speckle to achieve a varied surface texture. This layer is composed of a white uniform painting, on which a spread of a black micro-droplets is projected (Figure 4a,d). The displacement field is measured using VIC-2D software. The digital image correlation is performed with subsets as small as 15 pixels with 5 pixel steps. The physical dimension of one pixel is 166 µm.

## 3. Results and Discussion

**Figure 5:**
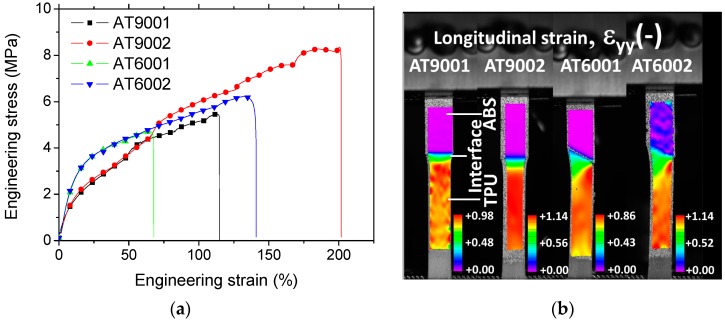
(**a**) Tensile response of 3D printed multi-material specimens as function of interface orientation and number of intertwining droplets and related (**b**) longitudinal strain fields derived from digital image correlation at a load level of 50% in length increase.

Figure 5a shows the tensile response of ABS/TPU samples manufactured using droplet-based 3D printing. These samples are differentiated according to the nomenclature introduced in the previous section. All samples demonstrate a typical elasto-plastic and damage behaviour. The damage component of the tensile responses can be read from the sudden change in stress magnitudes. In addition, there seems to be a coupling between damage and plasticity for all cases. Up to an engineering strain of 63%, the upper trends are those involving an ABS/TPU interface inclined by 60° with respect to the loading direction (AT6001 and AT6002). Within this load range, the observed low performance of specimens (AT9001 and AT9002) suggests that interfaces with exclusive normal traction at the interface involve low interfacial stiffness, which in turn affects the slope of the tensile response. This observation matches the expected effect related to the change of intersection angle at the interface (Figure 2). However, the modulation of the number of intertwining droplets does not match the expected result on interfacial stiffness. Examination of samples after loading shows characteristics of interfacial rupture. This means that there is a direct correlation between the ultimate properties (elongation at break, ultimate tensile strength) and the interfacial toughness. The largest ultimate properties are obtained for samples having two intertwining droplets. This demonstrates that the interface orientation plays a secondary role in tailoring the rupture properties compared to the number of intertwining droplets (AT6002 and AT9002). This means also that the observed effect of intertwining partially matches the description provided in Figure 2, which can be summarised as follows: a beneficial effect on interfacial resistance and unpredictable effect on interfacial stiffness.

Figure 5b shows the observed longitudinal strain field of the studied specimens issued from digital image correlation at a particular loading level of 50%. Full sequences of deformed specimens including other strain field components, i.e., transverse and shear strain fields are provided as supplementary data. At the observed load level, all strain fields are compared in the plasticity stage. The window of DIC measurement covers almost the width of the sample and a significant part of the sample in the tensile direction. This large sample coverage is sufficient to capture the strain localisation across the interface. High local strain levels exceeding the macroscopic engineering strain are experienced by all samples. These attest for a significant strain localisation. Also, the stretching of the strain magnitudes is significant, as demonstrated by the relative large difference between the scale strain limits. It has to be mentioned here that the tested specimens are flat with no particular shoulder. Hence, the difference in section reduction between the phases can be misleading because it is similar to what can be expected from a tensile response of a dog bone specimen. The interpretation of the observed behaviour is related to the large difference in section reduction from each side of the specimen. Thus, the image sequence of the deformed specimens reveals the contrast between the stiff ABS on the top and the ductile TPU on the bottom. At this particular load level, a significant constriction from the TPU side is observed. This means that the engineering quantities used to rank the tensile response in Figure 5a induces a different result at large load levels compared to nominal counterparts. In addition, strain field derived from DIC appears more or less homogenous within each phase, making it impossible to clearly sort out any process induced effect, such as layer arrangement or porosity distribution. However, the abrupt change in strain levels across the interface is clearly pointed out for all conditions. The comparison between the local fields across the interface well shows that a more significant heterogeneity is depicted when the intersection angle is levelled at 60° in comparison to 90° (Figure 5b). This is attributed to the larger intersection length at the interface, which allows a more significant interfacial damage to extend from the lateral sides. Interfacial shearing plays also a significant role on damage onset and growth because the loading direction is misaligned with respect to the interface orientation (Figure 2). The extension over which the change in strain magnitude is observed cannot be appropriately captured at large load levels because of the constriction effect. The measurements performed at load levels below 1% reveal an extension of 3.15 ± 0.80 mm for AT90XX and only 1.30 ± 0.21 for AT60XX. This extension is significant compared to the scale of intertwining (0.4 mm). Another characteristic feature is the intermediate strain levels noticed at the interface. If an interface is weak, high strain levels should prevail. The result shown in Figure 5b can be interpreted as either a complete interfacial debonding or strong interface behaviour. Examination of the full deformation sequences shows that the second hypothesis is likely to be the real scenario providing the accuracy of the DIC measurement system.

**Figure 6:**
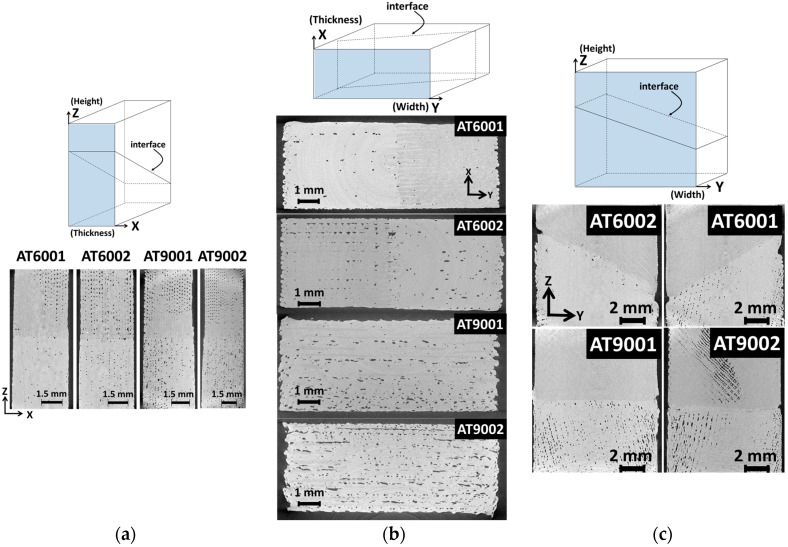
Cross-sections views at mid-depth from the tomograms of droplet-based printed slabs. (**a**) XZ; (**b**) XY; and (**c**) YZ. TPU appears as the lighter grey phase.

To understand better the microstructural implication of droplet-based printing process, and particularly the observed mechanical performance, X-ray micro-tomography investigation is considered. Cross-section views from different observation points are shown in Figure 6. These views represent the as-acquired grey level 2D images of the 3D stack prior image analysis treatment.

Figure 6a represents lateral views of all configurations where the interfacial arrangement along the thickness (X-direction, or building direction) and height (Z-direction) are highlighted. The inclination of the interface cannot be read from these views in the XZ plane.

**Figure 7:**
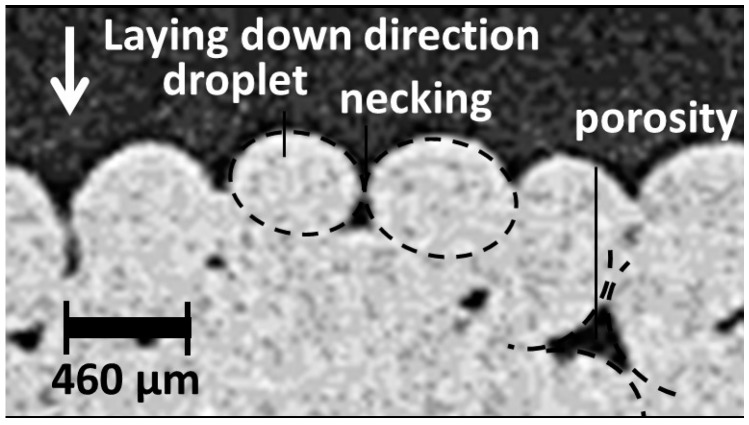
Illustration of the porosity genesis based on a local view of droplet arrangement. Due to the limited resolution of the magnified view, the contrast and sharpness were enhanced to reveal the main microstructural features.

However, any plane selected along the width shows the transition between the two phases, which is supposed to be an abrupt interface normal to Z-direction. In addition, the overall porosity arrangements and the intertwining behaviour at the interface are clearly depicted. For instance, porosities appear triangular in form and aligned in Z-direction forming a regular network, particularly in the upper part of the specimens. A zoom-in performed on a cross-section view provides some clue about the genesis of this particular form of porosity (Figure 7). Indeed, during the forming process, the laying down of contiguous droplets results in a necking phenomenon similarly to what is observed for a typical fused deposition modelling process [26]. The necking is a translation of a lack of cohesion between connected droplets during their cooling down. The original droplet form prior laying down, its surface tension and viscosity are all influential factors for the observed porosity form. The limited contact between droplets is also the genuine explanation for the observed surface roughness. Figure 7 suggests that the amount of overlapping between successive droplets can be improved to achieve a dense continuum of the deposited phase, particularly close to the sample boundaries. In general, the selection of the most appropriate overlapping ratio is a compromise between the printing duration and the density.

The comparison between the cross-section views in Figure 6a shows that there is some difference between the amounts of porosities. It appears, qualitatively, that a larger number of porosities is observed for a joint angle of 90°. In addition, despite the minor change in phase contrast between TPU (lighter grey levels) and ABS (darker grey levels), it appears that the amount of materials connecting each side of the interface depends significantly on the interface orientation. If we exclude the small tilts of the specimens with respect to image acquisition referential, the thickness of the interphase zone is unexpectedly larger than the one or two droplet size predefined as the printing conditions. Figure 6b shows another view in the XY plane of all considered configurations. This view confirms the large porosity content for joint angles of 90°. Therefore, there exists a relationship between the interface orientation and the amount of process-induced porosity. Here, one clearly sees the alignment of porosity channels in the Y-direction for AT9002 and to a lesser extent for AT9001. The observation of the porosity in Figure 6b reveals why the tensile response of AT9002 is not the lowest one despite the apparent large pore connectivity. This is because the relative orientation of the porosity channels in the lateral direction (Y-direction) prevents crack formation and extension by pore opening mechanism. A loading in the X direction would result, for example, in a lower mechanical response for AT9002. The porosity channels can be avoided if the kinematics of droplet positioning is modified, for example, by shifting the lateral position (Y). The overall content varies depending on both the printing orientation and the number of intertwining droplets at the interface. This view point proves the extent of material flowing from each side of the interface, especially for AT6001. Figure 6c shows the YZ views that capture the interface orientation. The interface appears relatively clean from any form of voids. However, significance of the porosity within each phase depends on the relative orientation of the droplet laying down with respect to the boundaries. The toolpath crossing at 45° generates lesser porosity for inclined interfaces at 60° compared to 90°.

This result reveals that the interface orientation with respect to part boundaries influences the process-induced porosity. These observations underline complex microstructural arrangement that needs to be further determined using a more qualitative analysis.

**Figure 8:**
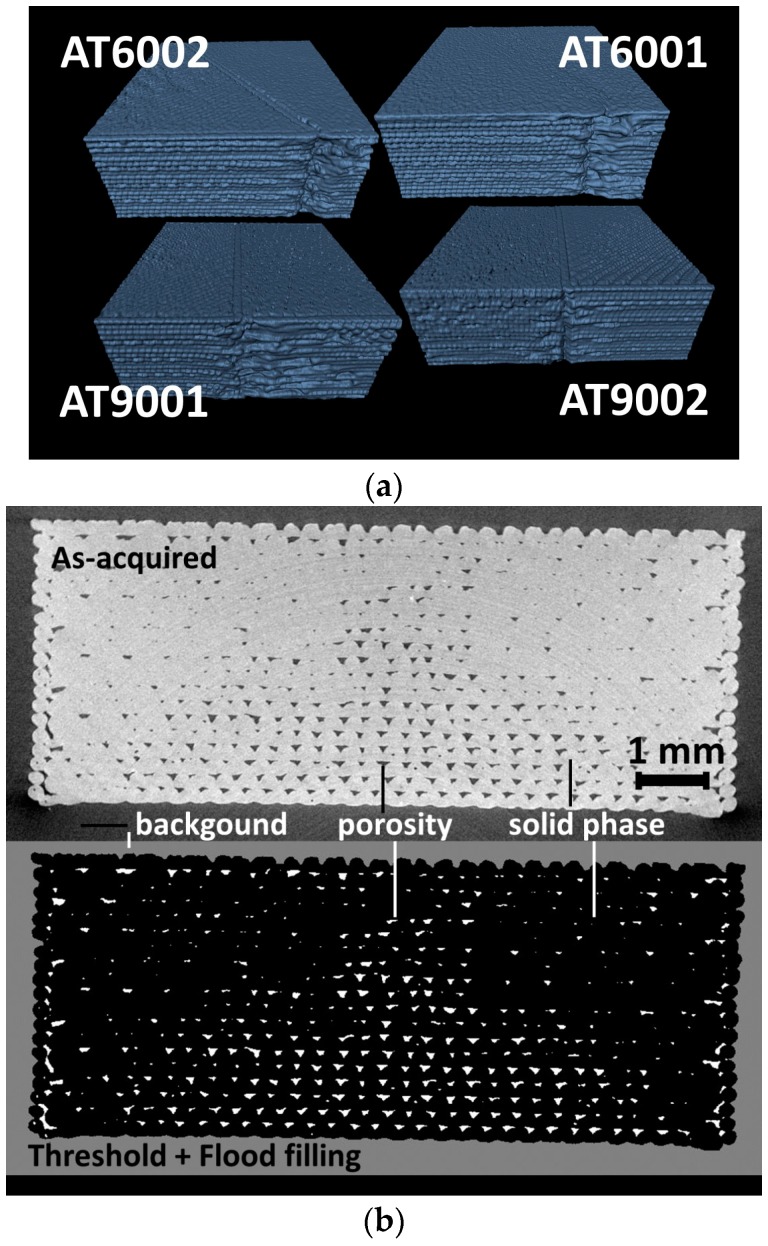
(**a**) Perspective views showing the surface topography of ABS/TPU slabs processed using a droplet-based 3D printing process; (**b**) Flood filling of the background to isolate the porous network from the external air.

The first explored feature is the surface topography. Figure 8a shows the overall envelope of all considered specimens, which details surface topography of the specimens near the interfacial junction between ABS and TPU. Three main features can be distinguished for all specimens: the building direction, the interface orientation, and the external frame. The building direction is parallel to the thickness of the specimens (X-direction, according to Figure 6).

Although the interface appears regular from the top view, the in-depth view shows a large perturbation in the droplet layering influenced by the change in material phase and successive trajectories. The overall structure of the rectangular frame used to confer structural stability is, as a consequence, altered at the junction between phases. The clean regular sample visually sensed turns to be microscopically similar to a notched configuration allowing departure of cracks under tensile loading. The surface of the printed slabs is also characterised by a regular motif of solidified droplets caused by the toolpath crossing in a sequence of +45°/−45°. A quantitative analysis of the porosity attributes such as morphology, connectivity and content can be introduced if the background sharing the same grey level with the porosity is discarded. To be as accurate as possible, the true volume of each slab is measured using a flood filling of the background. This technique illustrated in Figure 8b allows the determination of the exact nature of the porosity networks generated by droplet-based additive manufacturing. The particular good rendering of the flooding technique is achieved for all specimens because of the solid frame generated prior filling the specimen interior. This frame relatively isolates the internal porous structure from the surface.

**Figure 9:**
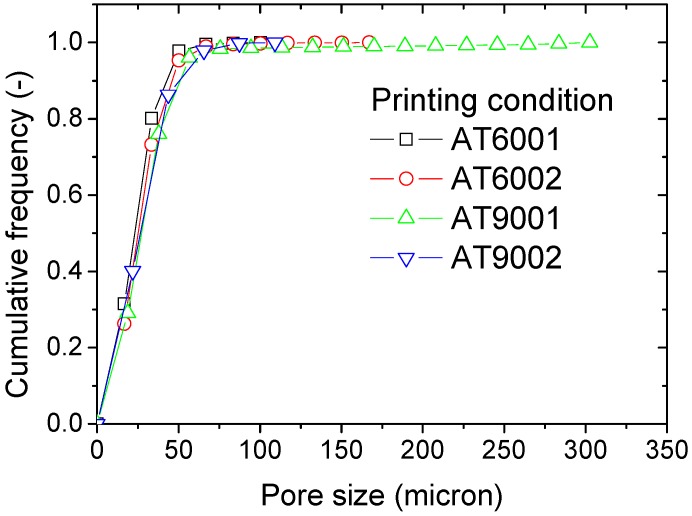
Cumulative plots of the porosity size distributions as function of printing conditions.

The size distribution of the porosities is the first examined feature. As stated in the former section, a 3D granulometry technique is used to determine the porosity size distribution. Figure 9 shows the cumulative representation of the porosity size distribution for all conditions.

The trends of porosity size are similar for all conditions, with the exception of the maximum pore size. The largest size class is found to substantially depend on the number of intertwining droplets and interface orientation. In addition, all distributions appear as unimodal because of the absence of an inflection point within the linear part. The cumulative form adopted in Figure 9 indicates the size range defining most of the porosity population. This range is, for all specimens, limited to 0–50 µm. Therefore, the porosities generated by the AM process can be considered as microscopic defects taking as a measurement scale their size. If their connectivity is taken, instead, as a scaling quantity, these defects should be qualified as macroscopic according to the observations in Figure 6.

A Gaussian profile is adopted to determine the centre and width of the size distribution, according to the expression:

(1) $$f\left(x\right)=y_{0}+\left(A/\left(w\sqrt{\pi /2}\right)\right)e^{-2\frac{\left(x-x_{c}\right)²}{w²}}$$

where f is the frequency at a particular size class x, $y_{0}$ is the ground frequency ($y_{0}=0$), $x_{c}$ is the centre position, w is the width of the distribution, and A is a constant.

The centre position $x_{c}$ derived from the fitting results can possibly differ from the average value if the distribution of the porosity size is not fully symmetrical. To account for such a possibility, the average porosity size is directly measured from the reading of the granulometry result as follows:

(2) $$x_{m}\left(µm\right)=\overset{N}{\underset{i=1}{\sum }}n_{i}x_{i\;\;},\;\overset{N}{\underset{i=1}{\sum }}n_{i}=1$$

where $x_{i}$ is the porosity size of class i and $n_{i}$ is its associated frequency, and N is the total number of classes.

Table 2 summarises the results for all attempted conditions. In addition to the quantities introduced in Expression (1), the maximum porosity size $x_{f}$ is also shown.

Table 2 shows that there is a slight difference between the centre positions of the order of 4%. The average porosity size $x_{m}$ is, for all conditions, larger compared to the centre position $x_{c}$ but the difference can be stated as being minor (6%). The only meaningful difference is the maximum porosity size, which can triple depending on the printing conditions.

In addition to the porosity size distributions introduced earlier, other microstructural characteristics of the porosity network are further analysed.

**Figure 10:**
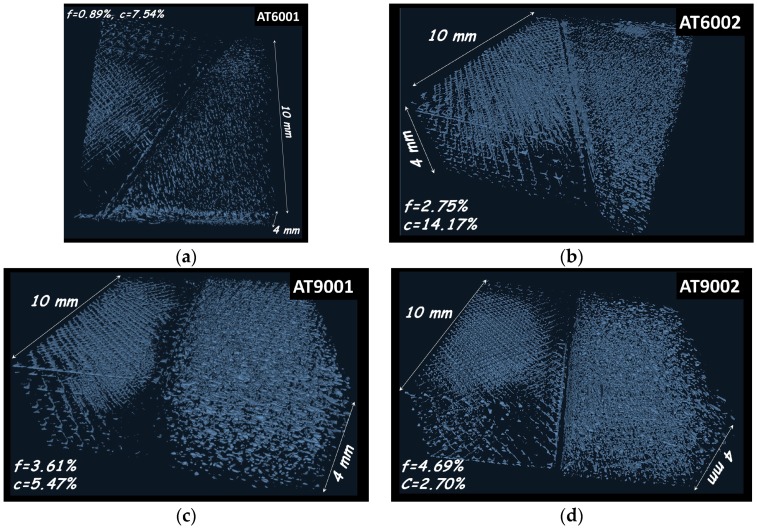
Porous network generated by droplet-based additive manufacturing as function of interface orientation and number of intertwining droplets. (**a**) 60°, one droplet; (**b**) 60°, two droplets; (**c**) 90°, one droplet; (**d**) 90°, two droplets. *f* is the porosity content and *c* is the pore connectivity.

Figure 10 compares the porosity networks achieved by varying the interface orientation and number of intertwining droplets. It should be mentioned that the granulometry technique used to derive the pore size distribution does not allow access to the pore connectivity. The porosity connectivity is measured using a 3D labelling technique, which isolates voxels belonging to the feature of interest depending on their ability to connect to the surrounding voxels. The pore connectivity is derived as the volume of the largest porosity $V_{f}$ feature with respect to the total volume $V_{t}$ of all labelled porosities.

(3) $$c\left(\%\right)=100\left(V_{f}/V_{t}\right)$$

where *c* is the porosity connectivity. Porosity connectivity and content for all conditions are given in Table 2.

In Figure 10, labels referring to the porosity content and pore connectivity are also provided. A regular porosity network is highlighted with a content varying between 1% and 5%. All the specimens share the same type of porosity network with more or less connected parts. This type of connectivity is genuine to the scheme adopted for the toolpath generation. Indeed, the tool path crossing with a sequence of +45°/−45° within the building plane is directly related to the form of the unit cell of the porosity network. One can clearly distinguish the orthogonality produced within the porous network in all space directions. A depletion zone free of any form of porosity at the interface indicates the presence of the solid rectangular frame. It is worth mentioning that the configurations achieving the lowest porosity contents (AT6001 and AT6002) rank as the same as the stiffness performance in Figure 5. Analysis of the porosity results also indicates a negative effect of the number of intertwining droplets, where largest porosity contents are correlated to two intertwining droplets. However, this effect of the number of intertwining droplets does not lead to a distinctive mechanical response for load levels lower than 50% (Figure 5). Meaningful differences between specimens appear for larger loads, particularly at the rupture point (elongation at break,$\;\varepsilon_{f}$). These differences can be correlated to the porosity level (f) and connectivity (c) as follows:

(4) $$\varepsilon_{f}\left(\%\right)\approx 135-82/f\left(\%\right)+192/c\left(\%\right);\;\;\;R^{2}=0.81$$

Although, there is no linear correlation between fracture stress ($\sigma_{f}$) and elongation at break ($\varepsilon_{f}$), a similar expression is obtained:

(5) $$\sigma_{f}\left(MPa\right)\approx 5.64-1.65/f\left(\%\right)+6.96/c\left(\%\right);\;\;\;R^{2}=0.78$$

Expressions (4) and (5) suggest that the failure is negatively influenced by the porosity level and connectivity. The reading of this result cannot be possible from Figure 10 without the fitting analysis because of the simultaneous variation of porosity level and connectivity. The comparison between the specimen performances shows that their failure is activated at different threshold strain values (Figure 5a). Knowing that optical recording suggests that the rupture mode for all specimens is interfacial debonding (Figure 5b), this means that differences in mechanical behaviour are attributed to different load transfers across the interface. The difference in load transfer is, in turn, influenced by the nature of the process-induced porous network. If the number of intertwining droplets is kept constant, a larger load transfer is achieved for an intersection angle at the interface of 90°. Therefore, the highest interfacial resistance is obtained here for an exclusive normal traction. Interfacial shearing associated with intersection angle of 60° contributes as a mechanism for the interfacial rupture because of the lower shearing component of the interfacial resistance. The interfacial shearing mode is expected to further enhance interfacial microcracking formation and coalescence because of the link that exists between this damage mechanism and the porosity characteristics revealed by the former expressions.

**Figure 11:**
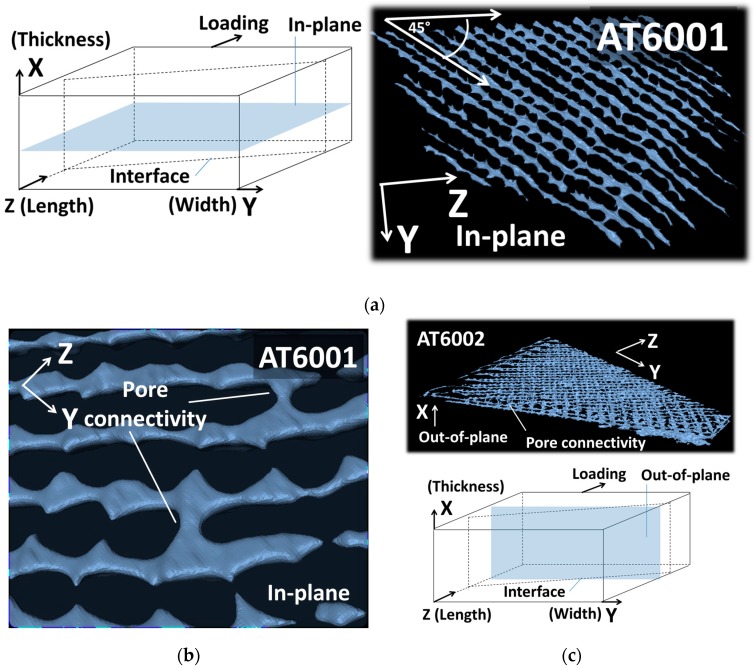

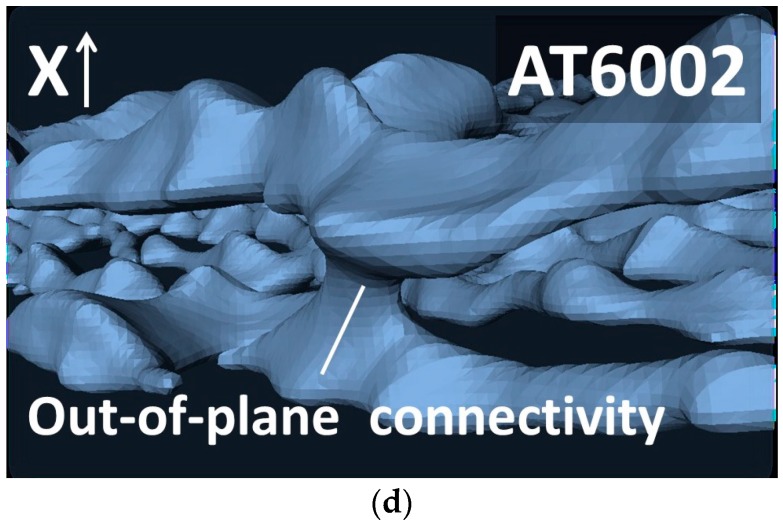
Largest connected porosities in (**a**) AT6001and (**b**) AT6002 illustrating two types of pore connectivity induced by droplet-based printing: (**c**) in-plane and (**d**) out-of-plane, i.e., parallel to the building direction.

Despite the demonstrated effect of porosity network on the mechanical performance, the overall analysis of the pore connectivity shows that none of the specimen demonstrates strong pore connectivity. Indeed, the largest connected porosity represents only 14% of the total porosity (AT6002). This result contrasts with pore connectivity induced by other additive manufacturing processes such as fused deposition modelling [25,27]. To understand better the type of connectivity generated by droplet-based process, Figure 11 shows the largest connected pore configurations isolated from the rest of the porous network for two conditions. The relative orientation of the largest connected pore with respect to the specimen main directions is added.

When the pore content is limited (<1% for AT60001), most of the pore connectivity occurs within the plane of construction (Figure 11a). Within this plane, pore connectivity is also anisotropic with one direction of major connectivity followed by limited density of porosities bridged in the orthogonal direction (Figure 11b). When the pore content increases (>2% for AT60002), out-of-plane pore connectivity appears, making it possible for successive layers of in-plane porosities to connect (Figure 8c). This connectivity is, however, limited and only minor bridges between these porosities are observed in the building direction (Figure 11d). This main characteristic of the droplet-based process to generate low pore connectivity is bounded by the fact that simple geometries are used. With the exception of the interface geometry, the orthogonal surfaces of the specimen do not allow much complexity to be expressed. We expect different extents of porosity connectivity to be generated from complex constructions involving overhanging parts and stair-stepping. Evaluation of this complexity is suggested in a future work based on the rendering of the double-cone geometry.

## 4. Conclusions

This study concludes that droplet-based additive manufacturing can lead to a successful printing experience of multi-material designs if interfacial properties are appropriately adjusted. The control of the interface quality in droplet-based additive manufacturing is a key distinctive feature over fused filament process. By adjusting the number of intertwining droplets, droplet-based additive manufacturing allows an efficient control of the ultimate performance of multi-material prints.

Because the stiffness performance of the 3D prints is found to depend on the orientation of the interface, it is also concluded that a varied local response is expected from the unbalanced interfacial shearing/tension behaviour. If the printed part is subjected to complex loads or if the load is simple but the part is complex, a careful selection of the assembling junction is recommended to allow a better distribution of the load along the interface. The equation of finding the proper junction profile in multi-material prints is not simple. This study also demonstrates that varied interface topography generates more porosity, that tends to connect. In general, the amount of porosity induced by the process significantly depends on the design complexity, the interface curvature and the quality of the interface.

Finally, the pore connectivity generated by the droplet-based process is found to be surprisingly low compared to filament-based processes. We expect that the cumulative effect of two discontinuities in filling the design would induce a more connected pore network. X-ray micro-tomography imaging demonstrates that limited porosity content of less than 5% result in only 14% of pore connectivity. The explanation for this performance probably lies in the droplet form, which is more convenient and efficient for filling gaps compared to filaments. A drastic change in pore connectivity can be achieved depending on the complexity of the part geometry and the cohesiveness of the droplet layers.

## Figures and Tables

**Table 1:** The main properties of feed materials used in droplet-based multi-material printing, according to the material sheet specifications. TPU: thermoplastic polyurethane; ABS: acrylonitrile butadiene styrene.

Nomenclature	TPU	ABS
Commercial name	Elastollan EC78A15	ABS Terluran GP35 Green
Grade	C78A	GP-35
Density (g/cm^3^)	1.18	1.04
Tensile strength (MPa)	50	-
Elongation at break (%)	650	12
Young’s modulus (MPa)	527	2300
Yield stress (MPa)	-	44
Flexural strength (MPa)	-	65

**Table 2:** Defect statistics for all printing conditions.

#	Porosity Size					Porosity Content and Connectivity	
	$x_{c}\left(µm\right)$	w(µm)	$x_{f}\left(µm\right)$	$R^{2}\left(-\right)$	$x_{m}\left(µm\right)$	f(%)	c(%)
AT6001	33	28	100	0.95	32	0.89	7.54
AT6002	34	28	167	0.99	35	2.75	14.17
AT9001	37	32	302	0.99	40	3.61	5.47
AT9002	35	31	109	0.98	38	4.69	2.70

## Supplementary Materials

Videos illustrating the strain fields components derived from digital image correlations are available online at www.mdpi.com/2073-4360/9/8/372/s1, Video S1: Evolution of longitudinal strain field, Video S2: Evolution of transverse strain field, Video S3: Evolution of shear strain field.

## References

[1] Gibson I., Rosen D.W., Stucker B. (2010). Additive Manufacturing Technologies.

[2] Zhai Y.W., Lados D.A., Lagoy J.L. (2014). Additive manufacturing: Making imagination the major limitation. Jom.

[3] Baumers M., Dickens P., Tuck C., Hague R. (2016). The cost of additive manufacturing: Machine productivity, economies of scale and technology-push. Technol. Forec. Soc. Chang..

[4] Berman B. (2012). 3-D printing: The new industrial revolution. Bus. Horiz..

[5] Mousa A.A., Bashir M.O. (2017). Additive manufacturing: A new industrial revolution—A review. J. Sci. Achiev..

[6] Singh R., Singh S. (2017). Additive manufacturing: An overview. Ref. Modul. Mater. Sci. Mater. Eng..

[7] Giffi C.A., Gangula B., Illinda P. (2014). 3D Opportunity in the Automotive Industry, Additive Manufacturing Hits the Road.

[8] Joshi S.C., Sheikh A.A. (2015). 3D printing in aerospace and its long-term sustainability. Virtual Phys. Prot..

[9] Peltola S.M., Melchels F.P.W., Grijpma D.W., Kellomäki M. (2009). A review of rapid prototyping techniques for tissue engineering purposes. Ann. Med..

[10] Krimi I., Lafhaj Z., Ducoulombier L. (2017). Prospective study on the integration of additive manufacturing to building industry—Case of a french construction company. Addit. Manuf..

[11] Weller C., Kleer R., Piller F.T. (2015). Economic implications of 3D printing: Market structure models in light of additive manufacturing revisited. Intern. J. Produc. Econ..

[12] Zanardini M., Bacchetti A., Zanoni S., Ashourpour M. (2016). Additive manufacturing applications in the domain of product service system: An empirical overview. Proc. CIRP.

[13] Gebler M., Schoot Uiterkamp A.J.M., Visser C. (2014). A global sustainability perspective on 3D printing technologies. Energy Policy.

[14] Hagiwara T. (2001). Recent progress of rapid prototyping photo-resin,“resin for stereolithography”. Macromolecular Symposia.

[15] Puebla K., Arcaute K., Quintana R., Wicker R.B. (2012). Effects of environmental conditions, aging, and build orientations on the mechanical properties of astm type I specimens manufactured via stereolithography. Rapid Prototyp. J..

[16] Dawoud M., Taha I., Ebeid S.J. (2016). Mechanical behaviour of ABS: An experimental study using fdm and injection moulding techniques. J. Manuf. Process..

[17] Paul R., Anand S. (2015). Optimization of layered manufacturing process for reducing form errors with minimal support structures. J. Manuf. Syst..

[18] Chua C.K., Wong C.H., Yeong W.Y. (2017). Roadmap on additive manufacturing standards. Standards, Quality Control, and Measurement Sciences in 3D Printing and Additive Manufacturing.

[19] Sun Q., Rizvi G.M., Bellehumeur C.T., Gu P. (2008). Effect of processing conditions on the bonding quality of fdm polymer filaments. Rapid Prototyp. J..

[20] Simchi A., Pohl H. (2003). Effects of laser sintering processing parameters on the microstructure and densification of iron powder. Mater. Sci. Eng. A.

[21] Thompson A., Maskery I., Leach R.K. (2016). X-ray computed tomography for additive manufacturing: A review. Meas. Sci. Technol..

[22] Guessasma S., Benseddiq N., Lourdin D. (2010). Effective young’s modulus of biopolymer composites with imperfect interface. Int. J. Solids Struct..

[23] Hbib M., Guessasma S., Bassir D., Benseddiq N. (2011). Interfacial damage in biopolymer composites reinforced using hemp fibres: Finite element simulation and experimental investigation. Compos. Sci. Technol..

[24] Guessasma S., Nouri H. (2015). Compression behaviour of bread crumb up to densification investigated using X-ray tomography and finite element computation. Food Res. Int..

[25] Nouri H., Guessasma S., Belhabib S. (2016). Structural imperfections in additive manufacturing perceived from the X-ray micro-tomography perspective. J. Mater. Proces. Technol..

[26] Guessasma S., Belhabib S., Nouri H., Ben Hassana O. (2016). Anisotropic damage inferred to 3D printed polymers using fused deposition modelling and subject to severe compression. Eur. Polym. J..

[27] Guessasma S., Belhabib S., Nouri H. (2015). Significance of pore percolation to drive anisotropic effects of 3D printed polymers revealed with X-ray μ-tomography and finite element computation. Polymer.

